# Risk Mapping of *Anopheles gambiae s.l.* Densities Using Remotely-Sensed Environmental and Meteorological Data in an Urban Area: Dakar, Senegal

**DOI:** 10.1371/journal.pone.0050674

**Published:** 2012-11-30

**Authors:** Vanessa Machault, Cécile Vignolles, Frédéric Pagès, Libasse Gadiaga, Yves M. Tourre, Abdoulaye Gaye, Cheikh Sokhna, Jean-François Trape, Jean-Pierre Lacaux, Christophe Rogier

**Affiliations:** 1 Département d’Infectiologie de Terrain - Equipe 7 “Maladies émergentes et moustiques”/Unité de Recherche sur les Maladies Infectieuses et Tropicales Emergentes (URMITE) - UMR6236, Institut de Recherche Biomédicale des Armées (IRBA), Marseille, France; 2 Laboratoire d’Aérologie, Observatoire Midi-Pyrénées (OMP), Université Paul Sabatier, Toulouse, France; 3 Direction de la Stratégie et des Programmes/Terre-Environnement-Climat, Centre National d’Etudes Spatiales (CNES), Toulouse, France; 4 Unité de Paludologie Afrotropicale - Equipe 7 “Maladies émergentes et moustiques”/Unité de Recherche sur les Maladies Infectieuses et Tropicales Emergentes (URMITE) - UMR6236, Institut de Recherche pour le Développement (IRD), Dakar, Sénégal; 5 Direction de la Climatologie, METEO-France, Toulouse, France; 6 Lamont-Doherty Earth Observatory (LDEO) of Columbia University, Palisades, New York, United States of America; 7 Institut Pasteur de Madagascar, Antananarivo, Madagascar; Johns Hopkins University, United States of America

## Abstract

**Introduction:**

High malaria transmission heterogeneity in an urban environment is basically due to the complex distribution of *Anopheles* larval habitats, sources of vectors. Understanding 1) the meteorological and ecological factors associated with differential larvae spatio-temporal distribution and 2) the vectors dynamic, both may lead to improving malaria control measures with remote sensing and high resolution data as key components. In this study a robust operational methodology for entomological malaria predictive risk maps in urban settings is developed.

**Methods:**

The Tele-epidemiology approach, *i.e.,* 1) intensive ground measurements (*Anopheles* larval habitats and Human Biting Rate, or HBR), 2) selection of the most appropriate satellite data (for mapping and extracting environmental and meteorological information), and 3) use of statistical models taking into account the spatio-temporal data variability has been applied in Dakar, Senegal.

**Results:**

First step was to detect all water bodies in Dakar. Secondly, environmental and meteorological conditions in the vicinity of water bodies favoring the presence of *Anopheles gambiae s.l.* larvae were added. Then relationship between the predicted larval production and the field measured HBR was identified, in order to generate *An. gambiae s.l.* HBR high resolution maps (daily, 10-m pixel in space).

**Discussion and Conclusion:**

A robust operational methodology for dynamic entomological malaria predictive risk maps in an urban setting includes spatio-temporal variability of *An. gambiae s.l.* larval habitats and *An. gambiae s.l.* HBR. The resulting risk maps are first examples of high resolution products which can be included in an operational warning and targeting system for the implementation of vector control measures.

## Introduction

Malaria is caused by a *Plasmodium* parasite transmitted among humans by the female mosquitoes/vectors of the *Anopheles* genu*s.* The location of the vectors larval habitats and their dynamics are the primary determinants of the spatial and temporal distribution of malaria transmission. Transmission units are defined as the systems where the larval habitats are the sources for the pathogen transmission [1]. Indeed, mosquitoes emerging from larval habitats have a somewhat limited flying range which depends on environmental conditions. Malaria risk is thus heterogeneous in space and time, as it is driven by the vicinity and dynamics of the larval habitats. Risk is further weighted by the dispersion and survival rates of adult mosquitoes, the availability of *Plasmodium* reservoir and the human vulnerability (which depends on availability of preventive devices as well as acquired individual immunity). In general, the number of larval habitats is more limited in cities were blood meal sources are more abundant, leading to lower vectors dispersal (a few hundreds of meters) in highly-populated urban settings [2], [3], [4], [5], [6] compared to open rural areas, where dispersal may reach several kilometers [7]. Consequently malaria transmission heterogeneity is higher within cities and may vary from district to district like in Brazzaville [8] or Dakar [2], [9].

It is well known that environment, climate (mainly rainfall amount and distribution, and temperature) and human activities [10], [11], [12], [13], [14] play an important role in determining the vector distribution [15], [16], [17], [18] and malaria epidemiology [19], [20]. A better understanding of the contribution of those ecological factors on the spatio-temporal dynamics of mosquitoes associated with malaria may lead to improvement of controlling measures. Indeed, environmental and water management, introduction of predators, use of larvicides, insecticide spraying or use of impregnated bed-nets all proved to be useful for an on-going fight against the disease [21], [22], [23], [24], [25], [26], [27], [28]. Nevertheless limited resources imply to focus the actions in places and time where and when they are the most useful. It has been argued that remote sensing technology has become a pre-requisite tool to assess the malaria burden, to model its spatio-temporal distribution, and plan effectively malaria control. The latter are key elements to implement within an operational system and thus to facilitate overall real-time monitoring of human health [29].

Urbanization occurs at a rapid pace and the United Nations predicts that by 2030, almost 60% of the world population will live in cities [30]. In consequence, urban malaria is nowadays an emergent public health issue that has been taken into account in the Roll Back Malaria (RBM) objectives, as demonstrated for example with the Rapid Urban Malaria Appraisal (RUMA) in sub-Saharan Africa [31]. Indeed, even if the availability of larval habitats and the mosquito survival may be reduced due to unfavourable environmental conditions, transmission of *Plasmodium* parasites has extensively been reported in cities, [32], [33]. The factors that impact transmission in urban areas have been identified as land use (including urban farming [34]), demography, municipal initiatives, individual and household characteristics, economic level, degree of urbanization, vector control measures, access to health care, as well as climatic and topographic factors [33], [35] or adaptation of the vector to new or polluted larval habitats [36], [37], [38], [39].

In Dakar, the capital city of Senegal, some malaria cases are recognised to be autochthonous [40] and may be severe cases [41], [42]. Between 2007 to 2010, the Entomological Inoculation Rate (EIR) during the transmission season has been evaluated between 0 and 17.6 infected bites in several districts of Dakar and its close suburb [9], [43] and the mean parasite prevalence in 2008 has been measured at about 16% in 50 districts of the capital [44]. In consequence, malaria risk in Dakar should not be underestimated even if epidemiological reports confirm that the number of cases is decreasing in the country (41% reduction of confirmed cases form 2008 to 2009) [45]. The strategic plan defined by the Senegalese National Control Program for 2011–2015 aims at going towards pre-eliminating malaria in the country. It includes fighting malaria during pregnancy, diagnosing and treating cases, managing outbreaks, coordinating supplies, promoting health, implementing universal coverage of insecticide-treated bed nets but also actualizing larvae and adult mosquitoes control strategies [46].

Risk maps have been defined in [47] as “outcomes of models of disease transmission based on spatial and temporal data”, incorporating “to varying degrees, epidemiological, entomological, climatic and environmental information”, and they have been applied to numerous diseases for mapping a current situation or even anticipating outbreaks with Early Warning Systems (EWS) [13]. Even if most of the risk mapping has been undertaken at large scales, previous studies have attempted to map malaria risk on small scales within rural [48] or urban environment, such as in Dar-Es-Salaam, [49], Malindi and Kisumu [50], [51], Ouagadougou [52] or Adama [53]. A study towards malaria risk mapping has also been achieved in 1996 and 2007 in Dakar [54]. In the present study, it is thought that the conceptual approach (CA) of Tele-epidemiology [55] could be applied to spatio-temporal mapping of entomological malaria risk in urban settings. This CA has been developed and patented by the French Spatial Agency and its partners, and consists in monitoring and studying human and animal disease dynamics which are closely related to climate and environment variability: i) choice of appropriate satellite data and dynamical models are evaluated, ii) all results are assessed and double-checked with extensive in-situ measurements allowing for identification of key biological processes. The objective here was to develop a robust operational methodology to produce entomological malaria predictive risk maps. The whole methodology relied on predicted vector risk areas (obtained from Human Biting Rate, or HBR) which were centred in the vicinity of predicted *Anopheles gambiae s.l.* larval habitats.

## Materials and Methods

### Study Site

The city of Dakar (14°40′20′′ North, 17°25′22′′ West) is the capital of Senegal located in the Cape-Verde Peninsula. In 2008, the population was estimated to almost 2.5 millions inhabitants. The highest point peaks at 104 m above sea level. The sahelian climate is modified by the proximity of the Atlantic Ocean, and the summer rainy season (monsoon) lasts from June to November, with average temperatures between 24°C and 30°C and average rainfall of about 400 mm. During the cooler and drier season from December to May temperatures are between 19°C and 25°C. During the studied years of 2007, 2008 and 2009, the annual rainfall was of 178 mm, 510 mm and 565 mm respectively (data from the Senegalese Weather Bureau).

### Entomological and Environmental Data

**Figure 1:**
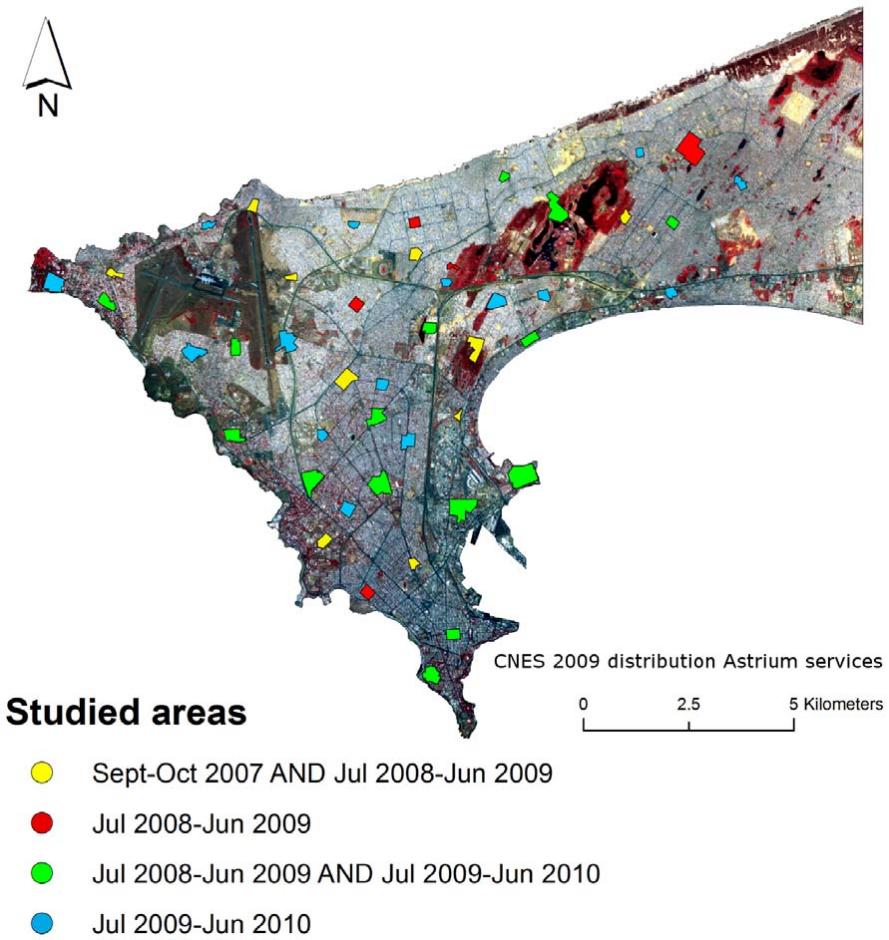
Spatial distribution of the 45 studied areas in Dakar and their period of study.

Entomological field studies have been undertaken during September-October of 2007 and from July 2008 to June 2010, and are detailed in [9], [43]. Briefly, the monitoring study was conducted from Dakar, Pikine, Thiaroye and Guediawaye sites, a total of 45 zones each covering a 200 m×200 m approximate area. They were chosen since they included as many diverse environments as possible in terms of type of urbanization, road network, vegetation, water and socio-economic level (Figure 1). Ten zones were studied for the September-October 2007 period, 30 zones for the July 2008 to June 2009 period, and 30 zones for the July 2009 to June 2010 period (named years 2007, 2008 and 2009 hereafter) for a total of 70 zone-years (one zone-year is a zone studied during one of the three studied periods). Adult mosquitoes sampling was carried-out approximately at the centre of the 200 m×200 m zones, once every other week using human landing catch, for a grand total of 3,096 person-nights of capture. For every studied zone, one catching point was located indoors and two were located outdoors except for Point E and Nord Foire were indoors locations could not have been found all year long. Among the 496,310 mosquitoes trapped, 9.06% (n = 44,967) were *Anopheles gambiae s.l*., 93.14% of which were *Anopheles arabiensis, 6.83% were Anopheles melas, 0.03% were Anopheles gambiae s.s. M form*. Extra species included *Anopheles pharoensis* (0.08%) and *Anopheles. ziemani* (0.03%). The HBR was expressed as the number of female *An. gambiae s.l.* bites per-person-per-night in a given area, and for a given date. Exhaustive search of all type of water bodies was undertaken every 10 days and for each zone. The latter were mapped using a Global Positioning System (GPS) device (5 m minimum precision) while physical, chemical and biological parameters (size, shade, floating and surrounding vegetation, water temperature, persistence during weeks) were recorded, including *Anopheles* larvae density using the standard dipping method and visual identification of larvae genera.

### Satellite Images

SPOT-5 (*Satellite Pour l’Observation de la Terre*) images [56] were programmed in order to be synchronous with the field work and were acquired during the three summer rainy seasons for the following dates: September 26, 2007, September 24, 2008, September 28, 2009, as well as during one dry season (May 11, 2009). For each date, data included three spectral bands at 2.5-m spatial resolution (green, red and near infrared - NIR). One band for the short wave infrared (SWIR) was also available with a 10-m spatial resolution. This band was downscaled at 2.5 m and stacked with the other three 2.5 m bands. A coastline mask was digitized and applied to all images, whereas inadvertent clouds were masked manually on each image. Clouds and their shadows covered 3.8 km^2^ on the 2007 image, 19.5 km^2^ in 2008 and 9.8 km^2^ of the September 2009 image, hiding parts of some studied zones. No clouds were detected with the May 2009 SPOT image. Since comparisons of multi-date images could be impeded by differences in atmospheric conditions, internal average relative reflectance (IARR) calibration - a classic normalization procedure - was undertaken for all images [57], [58].

A Digital Elevation Model (DEM) at 90-m spatial resolution was available from the Shuttle Radar Topography Mission (SRTM version 4.1) [59], [60].

Decadal Moderate Resolution Imaging Spectroradiometer (MODIS-Terra) images were available at 1-km spatial resolution for the full duration of the field work.

All images were chosen with the help of the published review [29] and each image processing was done using ENVI 4.7 (Exelis Visual Information Solutions) and the MODIS Reprojection Tool [61].

### Environmental and Meteorological Indicators

Several humidity, vegetation and soil indicators that were potentially associated with the presence or not of water, and the presence or not of *Anopheles gambiae s.l.* larvae and adults were calculated from each 4 bands SPOT image at 2.5-m spatial resolution, following [29]. Table 1 [62], [63], [64], [65], [66] displays parameters that were finally retained for the risk mapping.

**Table 1:** Environmental indicators calculated from the 4-bands SPOT-5 image at 2.5 m spatial resolution and potentially associated with the presence of water or the presence of *An.gambiae s.l.*

Environmental indicator	Spectral bands combination*	Description	Ref.
**NDVI (**Normalized Difference Vegetation Index)	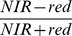	A value superior to 0.2 usually corresponds to a vegetated area, which gets denser when this value rises. Negative values indicate non-vegetated features such as barren surfaces (rocks and soils), water, built-up areas or asphalt.	62, 63
**NDWI Mac Feeters (**Normalized Difference Water Index)	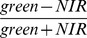	It delineates open water features while eliminating the presence of soil and terrestrial vegetation features. Its value increases with the presence of water and decreases with the presence of vegetation. It is also suggested that it may provide turbidity estimations of water bodies.	64
**MNDWI (**Modified NDWI Mac Feeters Index)	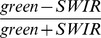	It can enhance open water features detection while efficiently suppressing and even removing built-up land noise as well as vegetation and soil noise.	65
**BI (**Brightness Index)	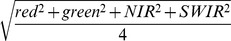	It characterizes soil physical properties, roughness, compactness or moisture content. High values correspond to natural or anthropogenic bared soils, without vegetation.	66

* NIR : Near infrared, SWIR : Short wave infrared.

An unsupervised isodata classification produced a map of Land Use and Land Cover (LULC) from the dry season SPOT image at 2.5 m. Validation of the classification quality was assessed using photo-interpretation by local experts. The final classification included six land-cover types identified as building, asphalt, sand, water, vegetation, bared soils (mixed or not with sparse vegetation) areas.

The DEM provided altitude information on Dakar and its suburbs at 90-m spatial resolution.

The freely available MODIS Reprojection Tool was used to extract day and night Land Surface Temperatures (LST) from MODIS images and to reproject the resulting images in UTM zone 28, WGS 84 datum. The LST was averaged for the Cape-Verde Peninsula and smoothed by calculating for each decade the averaged values from 3 decades. The daily values were deducted linearly between each decadal data, as a unique value for all studied zones.

Ground measurements of daily rainfall event were available from the Senegalese Weather Bureau for years 2007, 2008 and 2009. They were obtained at the Leopold Sedar Senghor International Airport (14°44′42′′N, 17°29′20′′W).

### Geographic Information System (GIS) and Statistical Analysis

A GIS was built within ArcGIS 9.2 (Environmental Research Systems Institute, Redlands, CA, USA) and statistical analyses were performed using Stata 11 (Stata Corporation, College Station, Texas). All ground information (water bodies and larval habitats, HBR, rainfall) was included in the GIS whilst processed images (indicators, LULC, LST, altitude) were added as geo-referenced layers.

Regressions between environmental and meteorological variables and presence of water or not (Step 1, logistic regression), presence of *Anopheles gambiae s.l.* larvae or not (Step 2, logistic regression) and *Anopheles gambiae s.l.* HBR (Step 3, negative binomial regression), were fitted using all environmental and meteorological indicators as explanatory variables. For each model, the variables with p-values <0.25 from univariate analyses were retained for multivariate analyses. In the case of correlated explanatory variables, the univariate model minimizing the AIC (Akaike information criterion) and having the best biological justification was selected. A backward stepwise selection procedure was applied in the final model to keep variables with p-values <0.05. The sampling scheme implied that some correlations could exist between observations obtained in a same zone or a same water body since nearby observations could be influenced by similar environmental factors. Thus, a random effect that could account for this type of autocorrelation was added to the models. In addition to the global models fitted with 3-years data, all models, or yearly sub-models, were also fitted with 2008 and 2009 yearly sub-samples of data. At steps 1 and 2, the large amount of observations allowed adjusting models using 80% of observations and assessing their validity with the remaining 20%. Models validity was assessed using ROC (Receiver Operating Characteristic) curve [67] (*i.e.,* representation of sensitivity against 1-specificity, or true positive rate versus false positive rate, providing the discriminative value of a test) for logistic models and using the Spearman rank correlation coefficient for the negative binomial models. The inversion and extrapolation of the models allowed drawing the several resulting maps in the Cape-Verde Peninsula.

Spatial autocorrelations between entomological observations were investigated for each year using the Moran’s I index calculated using ArcGIS.

### Detection of Water Bodies (Step 1)

As the basic requirement for the presence of mosquitoes is the availability of water bodies, the presence of latter was recorded in-situ and extrapolated for the whole city using the model adjusted during this first step. In the SIG, a 10-m grid was overlaid to the whole studied area, and the water bodies recorded at any date during each yearly ground study were included. Any grid square including a water body (even smaller than the pixel size), was set to 1, if not to 0, accounting for the dependent variable. Then, the mean values of every environmental indicator, as well as the surface of every LULC class and the altitude were extracted at the 10-m grid scale, for each year separately, representing the pool of explanatory variables to include in the univariate analysis. Using a 10-m grid allowed to improve calculation speed and to take into account uncertainty of the ground measurements (including uncertainties of the GPS receiver of about 5 m), while keeping the accuracy of using spatial information at 2.5-m spatial resolution. The inversion of the global 3-years model and the extrapolation to the whole Dakar peninsula was applied to generate three yearly 10-m resolution maps of the probabilities of presence of water bodies. The choice of a unique cut-off value (based upon the examination of the global ROC curve) was done for maximizing specificity *i.e.* minimizing the false positives. Its application provided raster maps of the presence/absence of water for each year. A closing morphology filter (dilatation followed by erosion using a structural element of 3×3 windows of 10-m pixels) eliminated small void without eliminating small water bodies. The resulting maps were vectorized with the option “polygon simplification” that smoothes the angles, meaning that every water body was assimilated as a single object.

### From Water Bodies to *An. gambiae s.l.* Larval Habitats (Step 2)

This second step aimed at estimating the probability of presence of larvae in the water bodies predicted during the first step. Environmental key factors for the presence of *Anopheles gambiae s.l.* larvae were isolated, and included into the preliminary map of water bodies. All the ground entomological observations (n = 2,051) associated with 170 water bodies were included in the analysis and the dependant variable was the presence/absence of larvae. The environment was taken into account as a mean of the ecological variables (indicators and LULC at 2.5-m spatial resolution) computed in and around (10-m ring buffer) the water bodies. This scale allowed taking into account the surface (*e.g.* surface cover as vegetation) and nearby structures (*e.g.* shade) on potential habitats that could impact on the presence/absence of larvae. Meteorological data were tested at different temporal scales until finding the best statistical association with the larvae presence, allowing for dynamical effects which may have an influence on larvae presence. The probability of presence of *Anopheles gambiae s.l.* larvae was predicted by computing the environmental and meteorological variables that were significantly associated in the 3-year global model for each of the water bodies mapped during step 1.

### From Larval Habitats to *Anopheles gambiae s.l.* HBR (Step 3)

Finally, based on the principle of the transmission units, relationship between the predicted larval production and the ground recorded *Anopheles gambiae s.l.* HBR was assessed. This allowed predicting the *Anopheles gambiae s.l.* HBR levels within the city, using the probability of presence of larvae and the surfaces of the corresponding larval habitats as the main source of adult mosquitoes, weighted by their daily survival and their dispersion distance. Indeed, a larval productivity surrogate was calculated as the sum of the probability of presence of larvae calculated during step 2 from 30 to 1 days before the actual adult catching night, since the daily vector survival rate is estimated at 82% in the Dakar peninsula [68]. Furthermore, the latter variable was summed-up in 200-m buffers and rings going from 300 m to 1,000 m of the catching points, the weight decreasing with distance This scale was derived from previous observations made in Pikine, where most of the *Anopheles* were caught at <285 m for the main larval habitat [2]. The extension to 1,000 m was undertaken for smoothing purposes and since maximum dispersal inside cities would not exceed 1 km. Dispersion depended on urban environment that was included in another variable as a the surfaces with buildings extracted from the classification image at 2.5 m, summed in buffers of 200 m and rings from 300 m to 1,000 m, with a weight decreasing with the distance from the catching point.

### Ethical Statement

Because Humans were not the subject of the research, no formal ethical clearance was required. Collectors carrying the human landing catches gave prior informed consent and received yellow fever immunizations and anti-malarial chemoprophylaxis for the duration of the study and one month afterward. For human landing catches undertaken in privately-owned locations, owners gave prior consent and were free to refuse the team entry to their house or garden at any time of the study, temporarily or permanently. The study did not involve endangered or protected species.

## Results

### Detection of Water Bodies (Step 1)

Step 1 was to detect water bodies in Dakar. Description of the distribution of the remotely-sensed variables in the 10-m grid cells is presented in Table S1a. Table 2 displays results from the logistic regression fitted to model the presence of water. Each observation (n = 48,858) corresponded to the presence/absence of water, based on the maximum water area recorded on the ground in the 45 studied zones during the 3 studied years, at 10-m resolution. In the global univariate and multivariate analysis, the SPOT-5 Modified Normalized Difference Water Index (NDWI) of the rainy season and the SPOT-5 NDVI of the dry season were positively associated with the presence of water while the SPOT-5 built-up area and the altitude from DEM were negatively associated. Predictions of the probability of presence of water for the validation sample (20% of all observations *i.e.* 9,772 observations) allowed calculating the area under the ROC curve at 0.86 (95% confidence interval: 0.85–0.88). Table S2 provides the results from the sub-models for years 2008 and 2009. The four variables remained significant in the yearly multivariate models. Even if the 95% confidence intervals obtained in the four models were not all overlapping, the direction and level of the regression coefficients remained similar. The areas under the ROC curve calculated after fitting the global model for the 2008 and 2009 years validation sub-samples were 0.83 (0.80–0.85) and 0.88 (0.87–0.90), respectively.

**Table 2:** Remotely-sensed environmental factors significantly associated with the maximum presence of water bodies recorded on the ground, including 80% of the observations for years 2007, 2008 and 2009 (logistic regressions with studied zone random effect are given - step 1).

	Univariate**			Multivariate		
80% of observations = 39,086 10 m grid squares (45 zones)	Coef.	95% CI*	p-value	Coef.	95% CI*	p-value
**SPOT MNDWI rainy season (mean)**						
Per 0.1 unit increase	0.87	0.83; 0.91	<0.001	1.07	1.02; 1.12	<0.001
**SPOT NDVI dry season (mean)**						
Per 0.1 unit increase	1.26	1.11; 1.41	<0.001	0.93	0.87; 0.98	<0.001
**SPOT built-up areas**						
Per 2.5 m pixel increase	−0.17	−0.18; −0.16	<0.001	−0.10	−0.11; −0.09	<0.001
**DEM elevation**						
Per meter increase	−0.24	−0.25; −0.22	<0.001	−0.15	−0.17; −0.13	<0.001

* 95% confidence interval.

** Only the variables significantly associated in the multivariate model.

The final result was one map for the water bodies for each year, at 10-m spatial resolution. In 2007, 2008 and 2009, a total of 1,294 (8.05 km^2^, 6.7% of the total studied surface *i.e.* Dakar and suburbs), 2,862 (9.19 km^2^, 7.9% of the total surface) and 1,697 (9.32 km^2^, 8.2% of the total surface) water bodies were respectively predicted outside of areas masked for cloudiness.

### From Water Bodies to *An. gambiae s.l.* Larval Habitats (Step 2)

Step 2 highlighted the environmental and meteorological determinants of the presence of *An. gambiae s.l* larvae or not, recorded during the field work. Description of the distribution of the remotely-sensed and meteorological variables from the water bodies is provided in Table S1b. The results from the univariate and multivariate logistic regressions are presented in Table 3 (global 3-year model) and Table S3 (sub-models for individual years 2008 and 2009). In the global univariate and multivariate analysis, the SPOT-5 NDWI Mc Feeters and the SPOT-5 Soil Brightness Index (BI) of the dry season, as well as the current night MODIS LST and the total ground rainfall in the preceding 30 days were positively associated with the presence of larvae in the water bodies. Predictions of the probability of presence of larvae from the validation sample allowed calculating the area under the ROC curve at 0.71 (95% confidence interval: 0.66–0.76). The direction and level of the regression coefficients of the yearly models remained similar to the global model. The areas under the ROC curve calculated after the fitting of this global model on the 2008 and 2009 validation sub-samples were 0.75 (0.68–0.82) and 0.72 (0.64–0.81), respectively.

**Table 3:** Remotely-sensed and ground meteorological and environmental factors significantly associated with the presence of *An. gambiae s.l.* larvae in the water bodies recorded on the ground, including 80% of the observations in 2007, 2008 and 2009 (logistic regressions with water body random effect are given - step 2).

	Univariate**			Multivariate		
80% of water bodies = 140 (1,638 observations)	Coef.	95% CI*	p-value	Coef.	95% CI*	p-value
**SPOT NDWI Mc Feeters dry season** ***						
Per 0.1 unit increase	0.99	0.40; 1.57	0.001	0.67	0.08; 1.26	0.025
**SPOT Soil BI dry season** ***						
Per 0.1 unit increase	1.33	0.62; 2.03	<0.001	0.95	0.26; 1.64	0.007
**MODIS current night LST**						
Per °C increase	0.28	0.21; 0.35	<0.001	0.13	0.05; 0.21	0.002
**Rainfall amount in the preceding 30 days**						
Per 10 mm increase	0.08	0.07; 0.09	<0.001	0.05	0.04; 0.07	<0.001

* 95% confidence interval.

** Only the variables significantly associated in the multivariate model.

*** Mean in the water body and a 10-m ring around.

**Figure 2:**
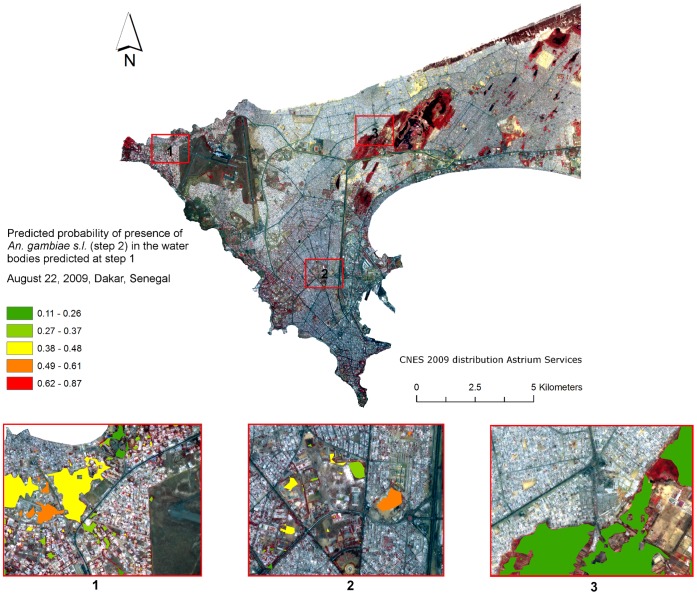
Predicted probability of presence of *An. gambiae s.l.* larvae (step 2) for August 22, 2009 in the water bodies predicted at step 1. Discretization was based on natural breaks.

The NDWI Mc Feeters and the Soil BI, as both environmental explanatory variables for the presence of larvae, were computed, using GIS, in and around each single water body predicted during the step 1. For every single day of the full duration of the study, including daily LST and rainfall, the inversion of the multivariate global model allowed drawing daily maps of the probabilities of presence of *An.gambiae s.l.* larvae in water bodies. Those daily maps could further be composited weekly, monthly or yearly to provide estimates of the urban global larval risk level at different time scales. Figure 2 provides blow-up images of the predicted probability of presence of *An. gambiae s.l.* in three districts of Dakar, calculated at the level of the water bodies predicted at step 1.

### From Larval Habitats to *Anopheles gambiae s.l.* HBR (Step 3)

Step 3 allowed defining the relationship between the predicted larval productivity surrogate and the field measured HBR, in order to generate *An.gambiae s.l.* HBR maps. Description of the distribution of the explanatory variables is presented in Table S1c. Results of the statistical analysis aiming at modelling the number of adult *An.gambiae s.l.* mosquitoes caught on human baits at a given date (averaged for the three catching points) are presented in Table 4 (global model) and Table S4 (yearly sub-models). Among the 70 zone-years, 61 were without cloud cover, for a total of 854 observations. A zone-year contained all the observations for a given zone, during one studied period. In the global univariate and multivariate analysis, the *Anopheles* larval productivity surrogate and the ground rainfall in the preceding 7 days were positively associated with the HRB whereas the SPOT-5 built-up and asphalt surface was a protective factor.

**Table 4:** *An. gambiae s.l.* larval productivity surrogate, and environmental and meteorological factors significantly associated with the *An. gambiae s.l.* HBR recorded in the 45 studied zones, including all the observations for years 2007, 2008 and 2009 (negative binomial regressions are given - step 3).

	Univariate**			Multivariate		
100% of adult catching points (61 zones/years, 854 observations)	Coef.	95% CI*	p-value	Coef.	95% CI*	p-value
***Anopheles*** ** larval productivity surrogate** ***						
Per unit increase	24.04	17.22; 30.86	<0.001	24.94	18.26; 31.62	<0.001
**Built-up and asphalt mean surface** ****						
Per m^2^ increase	−1.44	−1.92; −0.97	<0.001	−1.06	−1.46; −0.68	<0.001
**Rainfall amount in the preceding 7 days**						
Per 10 mm increase	0.15	0.09; 0.22	<0.001	0.16	0.10; 0.22	<0.001

* 95% confidence interval.

** Only the variables significantly associated in the multivariate model.

*** Sum of (probabilities of presence of *Anopheles* larvae x surfaces of larval habitats in km^2^) for all water bodies contained in the 200-m buffer and 300-m to 1,000-m rings around the catching points, weighted by the distance to the catching point.

**** Weighted with distance to catching point (from 200-m buffer to 300–1,000-m rings).

For the global model, the Spearman correlation coefficient calculated between the total adult *An. gambiae s.l.* count predictions and field measurements per zone-year (61 observations) was 0.75 (p<0.001). The Spearman coefficients calculated for each year separately were 0.72 (p<0.001) and 0.63 (p<0.001) for, 2008 and 2009, respectively.

**Figure 3:**
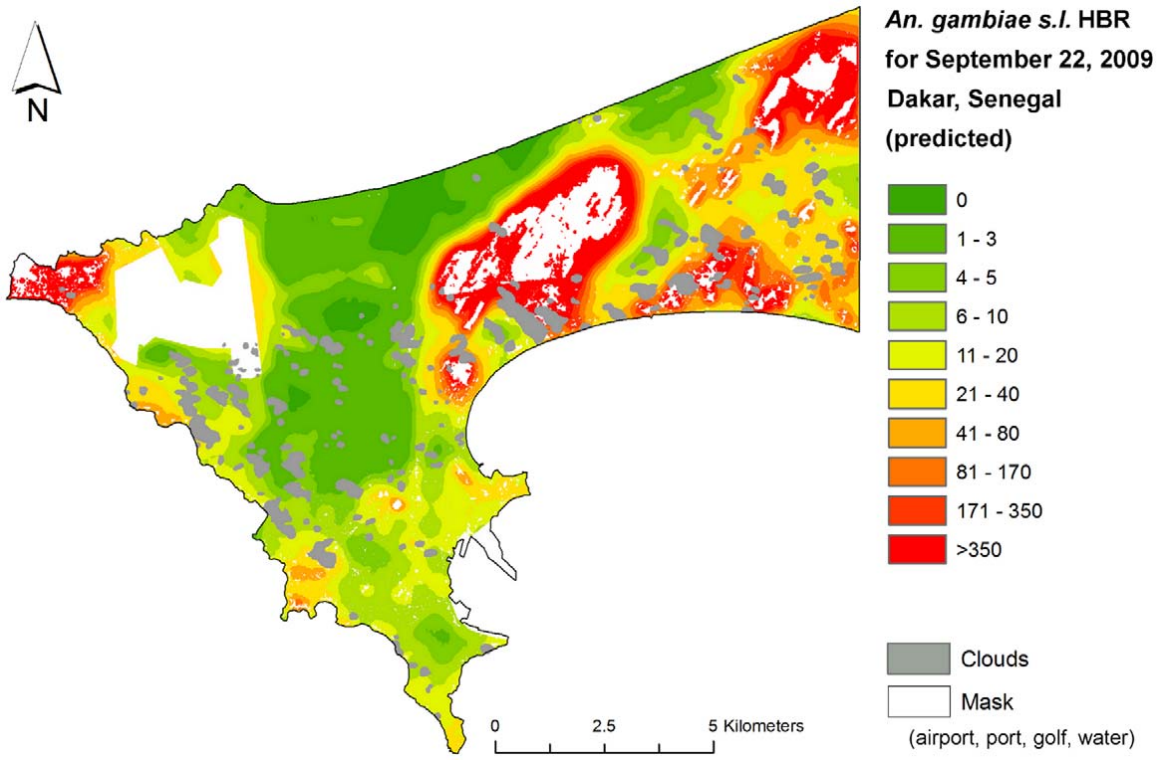
Predicted *An.gambiae s.l.* HBR (step 3, number of bites per person per night) for September 22, 2009. Discretization was based on HBR quantiles.

The inversion of the global model allowed the daily prediction of *An.gambiae s.l.* HBR at any location in Dakar with 10-m spatial resolution (Figure 3, example of predicted number of *An.gambiae s.l.* bites-per-person for September 22, 2009). The resulting maps were dynamics as the *Anopheles* HBR could be predicted on a day-by-day temporal basis.

### Spatial Autocorrelation

No spatial autocorrelation were detected among the HBR measured each year (Moran’s I Index  = 0.05 in 2007, −0.07 in 2008 and 0.05 in 2009). The patterns were neither clustered nor dispersed.

## Discussion

In the present study, the conceptual approach of Tele-epidemiology, developed by the French Spatial Agency and its partners [55] allowed to setup a robust operational methodology to draw high-resolution dynamic malaria entomological predictive risk maps in an urban setting. In addition this was done for two levels: risk maps of the *An. gambiae s.l.* larval habitats with their temporal larvae production level and dynamic risk maps of the levels of *An. gambiae s.l.* HBR. To our knowledge, these risk maps are first examples of entomological maps with high spatio-temporal scales. The latter could be included in an operational warning and targeting system. Indeed, for each step, the coefficients and validity of the models were similar for any of the single years, so the global model could be used for risk mapping for future years, based on updated satellite and meteorological information. Secondly, the maps are dynamics as they were built on a day-by-day basis.

Such maps have been successfully drawn in Dakar, Senegal. “The inclusion of the mechanistic link between malaria prevalence and vector habitat greatly improves the precision and accuracy of prevalence predictions” [69]. Indeed, modelling malaria risk requires first, to model the entomological risk. In the present study, a step-by-step mapping approach has been followed in order to closely relate modelling and the biological and physical mechanisms for the presence of water bodies, the larval development and for the adult survival and dispersion. The temporal resolution of larvae and adult *Anopheles* mapping was daily, allowing weekly, monthly and/or yearly maps production that could help in mosquitoes control within Dakar.

### Detection of Water Bodies

The result here was the production of 10-m resolution yearly maps for the potential water bodies in Dakar and suburbs. Mapping of water bodies was achieved using four predictors extracted from 2.5-m SPOT-5 images during the wet- and dry-seasons and from a 90-m DEM. First, the modified NDWI, an indicator suitable for enhancing and extracting water information [65], was logically positively associated with the presence of water bodies. Second, the NDVI of the dry season allowed mapping locations with permanent vegetation, being a proxy for the presence of water persisting all year long. Third, the increase of the areas with buildings (mapped at 2.5-m resolution) was negatively associated with the presence of water in the study unit (10-m grid squares). This was expected since the presence of surface water is not favoured by urbanization. This was also in adequacy with a longitudinal field study undertaken in Dakar in September 2009 (*i.e*. rainy season) in 355 private properties which showed that gardens, yards, flat roofs or balconies harboured very few water bodies [43]. Fourth, the presence of water bodies decreased with altitude, as already showed in the highlands in Kenya were low elevation was a risk factor for the presence of aquatic habitats [70]. In Dakar, even if orography is minimum, this was consistent with the water table near ground level in low altitude areas that can even be under the sea level.

The statistical models were adjusted to predict the water bodies present at any date of the ground prospecting for each year. This had the advantage to predict yearly maps of the maximum water bodies that could further be weighted depending on the rainfall and the type of water bodies. Event if the choice of the threshold for the presence of water has been done to maximize specificity in order to minimize the number of false positives, it is recognized that the detection of water could have been over-estimated in the Peninsula but in the scope of an operational use of risk maps, one could prefer not to underestimate the risk. Nevertheless, this choice could be refined depending on the needs for operational risk mapping. In addition, it was expected that some of the false positives will further be predicted with very low probability to harbour larvae and would then “disappear” from risk areas. It was also known that errors occurred during the water body mapping. In consequence, some large areas were manually masked around the port and the golf course but other confusions remained, mainly when discriminating water from dark asphalt. Whatever the indicator calculated or the classification process undertaken, black soils (*e.g*. parking areas) were sometimes recognized as water and this point should be particularly emphasized in order to improve water mapping accuracy.

In the present study, the pixels that were detected as water were grouped into water bodies as single objects, as it has been done in the North of Senegal in the mapping of the ponds harbouring larvae for Rift valley fever vectors [71], [72] or in Burkina Faso for malaria vectors [73]. Indeed, important predictors that could further be related to the presence of larvae, such as shade around the water body [43], were water body-related. In consequence, they had to be mapped at the level of the water body and not at the pixel-level. The latter could be relevant for large rural larval habitats [74] but has less biological meanings in urban settings where water bodies are smaller and may thus be more influenced by their surroundings. Here, an important fact is that the detection of water was derived from all types of water bodies, including the smaller ones as they are important breeding habitats for *An. gambiae s.l.* Indeed, any water body recorded on the ground and overlapping a 10-m pixel was included in the statistical analysis, even if their size was inferior to the pixel size. Consequently, even if the minimum size of a water bodt in the resulting map was 10 m×10 m, this could mean that only a fraction of the pixel was covered with water and that a smaller water body was included.

### From Water Bodies to *Anopheles gambiae s.l.* Larval Habitats

The results from this step were daily maps of the probabilities of presence of *An. gambiae s.l.* in the potential habitats mapped at the previous step. The larval habitats parameters that were recorded on the ground were used to understand the biological meaning of the remotely-sensed variables. But they were not assimilated into risk models, in the event of extrapolation of the models when no ground data is available.

The first significant 2.5-m SPOT-5 variable was the mean NDWI Mc Feeters (dry season) averaged in the water bodies and their 10 m surrounding that was positively associated with the presence of larvae. Comparison of the NDWI Mc Feeters with ground data of the present study indicated that higher values were related to temporary water bodies and water bodies with muddy bottom, while in [43], it has been showed that those two factors were associated with the presence of *Anopheles* larvae. These results were consistent with the known preference of *An. gambiae s.l.* for breeding in temporary pools [75], [76] as they are usually under sunnier conditions and may contain fewer predators [77]. Muddy bottom could be a proxy for an environment suitable for providing food for the development of larvae as well as for facilitating persistence of water. In addition, the NDWI Mc Feeters decreases with the presence of vegetation and even if data did not show any correlation between this indicator and shade, they may yet be related and their association could also lie on the unfavourable action of shady conditions.

The second significant 2.5-m SPOT-5 variable was the mean Soil BI of the dry season in the water bodies and their 10 m surrounding that was also positively associated with the presence of *An. gambiae s.l.* larvae. The explanation may be that BI was related to temporary water bodies, muddy bottom and water bodies outside of market-gardens, as recorded on the ground.,The field study [43] showed that, in addition to temporary water bodies and muddy bottom, a location outside of a market-garden was a risk factor for the presence of larvae. The negative association between the market-gardens and the larvae probably mainly lied in the frequent presence of larvivorous fishes in wells [43] that are effective predators [78], and in the frequent perturbations of water due to watering; however, one cannot exclude that the use of pesticides by urban farmers had lowered the presence of larvae [79], [80].

The first significant meteorological variable associated with the presence of *An. gambiae s.l.* larvae in the water bodies was the night MODIS LST. The entire development cycle duration from eggs to emerging adults decreases with temperature increase [81], consistent with the fact that LST was significantly positively associated with the presence of larvae. Indeed, it has been showed in the context of vector-borne diseases, that LST correlates well with the prevailing temperature of the air in Africa with +/−4°C [82]. Night LST was better associated than day temperature as it was probably less impacted by solar radiation and more representative of the averaged air temperature.

The second significant meteorological variable was the total rainfall amount during the 30 days prior to the actual ground larvae collection. On one hand, rainfall pattern drives the availability of surface water for mosquitoes breeding. At this point of the study, only flooded water bodies were included so rainfall was already implicitly taken into account. The rainfall amount could then be related to the persistence of water bodies. Indeed, the persistence of water bodies assessed from one field visit to another was significantly associated with the presence of *An. gambiae s.l.* larvae [43]. On the other hand, rainfall amount was a surrogate variable for the seasonal evolution which was highly related to the global larval productivity in the city. Lagged rainfall has already been associated with the presence of *Anopheles* densities in temporary or permanent breeding sites [83]. The inversion and the spatio-temporal extrapolation of the statistical model allowed probability prediction of presence of *An. gambiae s.l.* larvae for each water body that has been mapped during step 1, at any date of the follow-up.

### From Larval Habitats to *Anopheles gambiae s.l.* HBR

Daily maps of *An. gambiae s.l.* HBR at 10-m spatial resolution were produced. Based on the transmission units definition, the prediction of the presence of *Anopheles* vectors measured on the ground was achieved using: i) a larval productivity surrogate summed for all the water bodies predicted around the catching points, ii) the mean area of buildings and asphalt in the surroundings and iii) total rainfall during the 7 previous days. Although the EIR was available from the field study, it has been chosen to work with HBR. Indeed, the EIR is influenced by factors that are not only environmental (access to treatment, vectorial competence…) so the environmental and climatic modelling could have been biased. Nevertheless, in Dakar, no significant differences of infection rates of mosquitoes among different urban quarters could have been highlighted [9], [43]. Consequently, it can be considered that the actual risk of malaria transmission in Dakar is proportional to the HBR, wherever the districts.

The calculation of the larval productivity surrogate was based on the larval production, at a scale of 200 to 1,000 m, according to the transmission units definition. The 200 m scale was chosen as in Pikine, most of the *Anopheles* were caught at <285 m for the main larval habitat [2]. The extension to 1,000 m was undertaken for smoothing purposes and also because maximum dispersal in cities would not exceed 1 km. The biology of the *An. gambiae s.l.* vectors in an urban environment was also taken into account with the inclusion of daily survival rates (82%) that had been evaluated in Pikine, close to Dakar [68]. In consequence, the larval productivity surrogate was calculated as the sum of the probability of presence of larvae from 30 to 1 days before predicting the HBR, this period providing 100% of the mosquitoes that may bite at day 0.

Buildings and asphalt surfaces were unfavorable factors for the vector densities, in adequacy to the fact that sparsely built-up areas are known to be risk factors for malaria in cities [52]. First, the urban environment is unfavorable for the apparition of water bodies and this could partly modify any over-estimation that could have occurred at previous steps. Second, urbanization decreases the life span of the adult mosquitoes as they do not provide appropriate resting and feeding sites. Third, the high presence of buildings can be a proxy for a high human population density, leading to the dilution of the *Anopheles* bites. In Dakar, the urbanization has already been associated with entomological parameters. Indeed, a study showed that almost 60% of the variability in anopheline HBR measured in 1994–97 [84], [85] was explained with only one variable: the built-up area in a 300 m-radius buffer around the catching points [54]. Further investigation could improve the information provided by the built-up areas by adding information on land-use (residential areas, industries…) and human population densities.

The rainfall amount during the 7 days preceding the mosquito collection night introduced temporal information in the model. Indeed, as the water surface was constant over a year (step 1), the rainfall variable allowed taking into account the seasonal variations of the water availability in the HBR prediction. It also may have been associated with the adult mosquito survival, as a surrogate for humidity.

The extensive ground study concluded that malaria risk heterogeneity in Dakar was very high, according to the spatial and temporal distribution of vectors [9], [43]. The resulting risk maps confirmed this heterogeneity for the entire Peninsula, outside of the field studied zones.

### Malaria Control, Hazard and Vulnerability

In the present study, risk mapping has been undertaken at the entomological level and the risk maps could be seen as dynamical predictive tools for assisting vector control in space and time. Indeed, the presence of transmission units promotes targeted interventions as more effective than random control measures. Among the numerous facets of the integrated fight against malaria, vector control aims at reducing larval and adult *Anopheles* densities and an integrated vector management is recommended by the World health Organization (WHO) [86]: environmental (*e.g.* source reduction, waste management), mechanical (*e.g.* house improvement), biological (*e.g.* larvae predators) and chemical management (*e.g.* indoor residual spraying, larviciding, impregnated bed-nets), are relevant. Mapping the vector-borne diseases determinants has also been suggested as important [86]. Among those possibilities, larval control has been shown as being a powerful tool for malaria control having led to elimination in many historical examples [21], [25]. Limitating the number of larval habitats but also increasing the research duration for oviposition sites [87] can lead to a decrease in transmission [23]. Larvae risk maps, as a result from modelling, could guide this component of the integrated vector management measures as it has been modeled that a focus on the most productive breeding sites can lead to significant reductions, not only in adult mosquito productivity but also of the incidence and prevalence of malaria [88]. Those risk maps meet the WHO recommendations mentioning that “larviciding must be specially adapted to each locality and must be carried-out thoroughly and selectively” “(targeted at the most productive breeding sites)”, especially in urban areas where “it is most likely to be cost-effective”, and that “larviciding should be considered for malaria control (with or without other interventions) only in areas where the breeding sites are few, fixed and findable” [89]. In addition, it as had been showed that that malaria transmission can occur in Dakar both outdoors and indoors [43], long lasting impregnated bednets (LLINs) may not be sufficient for malaria vector control and larval control should be an important part of the control measures in the city. Vector risk maps of adult mosquitoes could help targeting mosquitoes control, since control of the adult stages also proved to be useful in several examples [27], [28]. As WHO also recommend to use indoor residual spraying (IRS) as part of the integrated vector control management [86], [89], this second level of risk maps may be used to target imago control activities. In Senegal, as the strategic plan defined by the Senegalese National Control Program for 2011–2015 aims at going towards pre-eliminating malaria in the country and actualizing larvae and adult mosquitoes control strategies [46], the availability of entomological risk maps could be of interest in the implementation of such revised strategies.

In addition to this entomological approach, it should be emphasized that malaria transmission occurs only where and when a competent infected vector meets a human sensitive population, if a *Plasmodium* reservoir is present. Thus, upcoming improved mapping should include immunological, parasitological, epidemiological and socio-economical data in addition to the entomological risk mapped in the present study. Furthermore, the precise definition of a risk area is a place where hazard and vulnerability must overlap. Hazard represents the “potential risk”, *e.g.,* the vector distribution, and vulnerability relates to the distribution, sensitivity and exposure of human populations. In consequence, urban population mapping is also a challenging objective, especially when no or poor population census report is available. In the present study, HBR has been mapped only for populated areas and large inhabited places have been manually removed (like airport fields and marshlands). Nevertheless, no socio-economic and behavioural information have been taken into account among the human population and it is expected that the inclusion of such information - *e.g.* type of housing, use of antivectorial devices - would weight the actual individual malaria risk.

### Conclusion

The Tele-epidemiology CA has been successfully applied for mapping entomological malaria risk in a sub-Saharan urban setting. Remotely-sensed environmental and meteorological data, associated with a large amount of ground entomological data collected specifically, allowed developing of a robust operational methodology to draw different levels of malaria entomological dynamic predictive risk maps that could be of interest for planning and targeting malaria control in urban settings. The first mapping level predicted the potential *An. gambiae s.l.* larval habitats in Dakar (depending upon the predicted presence of water, as well as environmental and meteorological conditions) that could guide local larval management. The second level was a map of the *An. gambiae s.l.* HBR (driven by surfaces and potential productivity of larval habitats as well as urbanization levels and rainfall amount) which could guide adult mosquitoes control activities. The results of the present study could be seen as providing the basic elements for real-time monitoring of human health. Within the framework of the EEOS-Malaria (Epidemiology Earth Observation Services - Malaria) project, effective operational malaria risk mapping is to be automated and implemented in other sub-Saharan cities.

## Supporting Information

Table S1 Description of the quantitative remotely-sensed and meteorological variables included as explicative variables in the three-step modelling (Table S1a for step 1, Table S1b for step 2, Table S1c for step 3)
(DOC)

Table S2 Remotely-sensed environmental factors significantly associated with the maximum presence of water bodies recorded on the ground, including 80% of the observations for years 2008 and 2009 separately (multivariate logistic regressions with studied zone random effect are given - step 1).
(DOC)

Table S3 Remotely-sensed and ground meteorological and environmental factors significantly associated with the presence of *An. gambiae s.l.* larvae in the water samplings recorded on the ground, including 80% of the water bodies in 2008 and 2009 separately (multivariate logistic regressions with water body random effect are given - step 2).
(DOC)

Table S4 *An. gambiae s.l.* larval productivity surrogate and environmental factors associated significantly with the *An. gambiae s.l.* HBR recorded on the ground, including all the observations for years 2008 and 2009 separately (negative binomial regressions are given - step 3).
(DOC)
