# Supplementary material for: Risk Mapping of Anopheles gambiae s.l. Densities Using Remotely-Sensed Environmental and Meteorological Data in an Urban Area: Dakar, Senegal
Source: PLoS One. 2012 Nov 30;7(11):e50674. doi: 10.1371/journal.pone.0050674 (PMC3511318; doi:10.1371/journal.pone.0050674)
Supplement: Table S1 — Description of the quantitative remotely-sensed and meteorological variables included as explicative variables in the three-step modelling (Table S1a for step 1, Table S1b for step 2, Table S1c for step 3) (DOC) [file pone.0050674.s001.doc]

Table S1. Description of the quantitative remotely-sensed and meteorological variables included as explicative variables in the three-step modelling (Table S1a for step 1, Table S1b for step 2, Table S1c for step 3).

Table S1a.

|  |  | Water present | Water absent |
| --- | --- | --- | --- |
|  | **n observations = 48,858** | **n=5,642** | **n=43,216** |
| **SPOT MNDWI rainy season** | Range | -0.22 ; 0.57 | -0.34 ; 0.41 |
|  | Mean in the 10 m grid cells | 0.09 | 0.01 |
|  | 95% CI* | 0.09 ; 0.10 | 0.01 ; 0.01 |
|  | 25-50-75 percentiles | 0.02 ; 0.08 ; 0.16 | -0.04 ; 0.00 ; 0.05 |
| **SPOT NDVI dry season** | Range | -0.13 ; 0.39 | -0.13 ; 0.40 |
|  | Mean in the 10 m grid cells | 0.07 | 0.00 |
|  | 95% CI* | 0.07 ; 0.07 | 0.00 ; 0.00 |
|  | 25-50-75 percentiles | -0.01 ; 0.04 ; 0.14 | -0.04 ; -0.02 ; 0.01 |
| **SPOT built-up areas (number of 2.5 m pixels in the 10 m units)** | Range | 0 ; 16 | 0 ; 16 |
|  | Mean in the 10 m grid cells | 1.17 | 4.75 |
|  | 95% CI* | 1.09 ; 1.25 | 4.70 ; 4.80 |
|  | 25-50-75 percentiles | 0 ; 0 ; 0 | 0 ; 2 ; 9 |
| **DEM elevation (m)** | Range | -2 ; 41 | -2 ; 49 |
|  | Mean in the 10 m grid cells | 8 | 18 |
|  | 95% CI* | 8 ; 9 | 18 ; 19 |
|  | 25-50-75 percentiles | 4 ; 7 ; 10 | 10 ; 16 ; 29 |

* 95% confidence interval

Table S1b.

|  |  | *An.gambiae s.l.* larvae present | *An.gambiae s.l.* larvae absent |
| --- | --- | --- | --- |
|  | **n observations = 2,051** | **n=739** | **n=1,312** |
| **SPOT NDWI Mc Feeters dry season **** | Range | -0.25 ; 0.08 | -0.26 ; 0.08 |
|  | Mean | -0.04 | -0.08 |
|  | 95% CI* | -0.05 ; -0.04 | -0.09 ; -0.08 |
|  | 25-50-75 percentiles | -0.07 ; -0.02 ; 0.00 | -0.15 ; -0.07 ; -0.02 |
| **SPOT Soil BI dry season **** | Range | 0.29 ; 0.64 | 0.29 ; 0.63 |
|  | Mean | 0.46 | 0.44 |
|  | 95% CI* | 0.46 ; 0.47 | 0.44 ; 0.44 |
|  | 25-50-75 percentiles | 0.42 ; 0.46 ; 0.51 | 0.41 ; 0.43 ; 0.46 |
| **MODIS current night LST (°C)** | Range | 16.8 ; 25.0 | 16.8 ; 25.0 |
|  | Mean | 21.9 | 20.9 |
|  | 95% CI* | 21.7 ; 22.0 | 20.8 ; 21.0 |
|  | 25-50-75 percentiles | 21.2 ; 21.8 ; 22.8 | 18.9 ; 21.2 ; 22.4 |
| **Ground rainfall amount in the preceding 30 days (mm)** | Range | 0 ; 342.7 | 0 ; 342.7 |
|  | Mean | 150.8 | 85.7 |
|  | 95% CI* | 142.7 ; 158.9 | 80.4 ; 91.0 |
|  | 25-50-75 percentiles | 48.0 ; 119.9 ; 240.6 | 0.0 ; 48.0 ; 125.6 |

* 95% confidence interval

** Mean in the water body and a 10-m ring around.

Table S1c.

|  |  | *An.gambiae s.l. HBR = 0* | *An.gambiae s.l. HBR > 0 and <2* | *An.gambiae s.l. HBR >= 2 and <12* | *An.gambiae s.l. HBR >= 12* |
| --- | --- | --- | --- | --- | --- |
|  | **n observations = 854** | ***n=380*** | ***n=157*** | ***n=154*** | ***n=163*** |
| ***An.gambiae s.l.* larval productivity surrogate **** | Range | 0.000 ; 0.071 | 0.000 ; 0.127 | 0.000 ; 0.192 | 0.000 ; 0.470 |
|  | Mean | 0.006 | 0.013 | 0.022 | 0.064 |
|  | 95% CI* | 0.005 ; 0.007 | 0.009 ; 0.016 | 0.017 ; 0.026 | 0.051 ; 0.077 |
|  | 25-50-75 percentiles | 0.000 : 0.001 ; 0.007 | 0.000 ; 0.003 ; 0.017 | 0.003 ; 0.010 ; 0.032 | 0.008 ; 0.032 ; 0.085 |
| **Built-up and asphalt mean surface ***** | Range | 1.09 ; 3.77 | 1.09 ; 3.77 | 1.09 ; 3.05 | 1.09 ; 2.51 |
|  | Mean | 2.23 | 2.03 | 1.87 | 1.72 |
|  | 95% CI* | 2.15 ; 2.31 | 1.94 ; 2.13 | 1.79 ; 1.95 | 1.65 ; 1.79 |
|  | 25-50-75 percentiles | 1.51 ; 2.20 ; 2.91 | 1.61 ; 2.08 ; 2.25 | 1.37 ; 1.99 ; 2.20 | 1.32 ; 1.90 ; 2.10 |
| **Rainfall amount in the preceding 7 days (mm)** | Range | 0.0 ; 110.1 | 0.0 ; 162.1 | 0.0 ; 162.1 | 0.0 ; 162.1 |
|  | Mean | 3.8 | 17.6 | 22.8 | 38.4 |
|  | 95% CI* | 2.3 ; 5.2 | 13.2 ; 22.0 | 17.5 ; 28.1 | 31.5 ; 45.3 |
|  | 25-50-75 percentiles | 0.0 ; 0.0 ; 0.0 | 0.0 ; 0.2 ; 33.6 | 0.0 ; 6.4 ; 35.0 | 0.0 ; 27.1 ; 60.5 |

* 95% confidence interval

** Sum of (probabilities of presence of *Anopheles* larvae x surfaces of larval habitats in km²) for all water bodies contained in the 200-m buffer and 300-m to 1,000-m rings around the catching points, weighted by the distance to the catching point.

*** Weighted with distance to catching point (from 200-m buffer to 300-1,000-m rings).
