# Supplementary material for: Risk Mapping of Anopheles gambiae s.l. Densities Using Remotely-Sensed Environmental and Meteorological Data in an Urban Area: Dakar, Senegal
Source: PLoS One. 2012 Nov 30;7(11):e50674. doi: 10.1371/journal.pone.0050674 (PMC3511318; doi:10.1371/journal.pone.0050674)
Supplement: Table S2 — Remotely-sensed environmental factors significantly associated with the maximum presence of water bodies recorded on the ground, including 80% of the observations for years 2008 and 2009 separately (multivariate logistic regressions with studied zone random effect are given - step 1). (DOC) [file pone.0050674.s002.doc]

Table S2. Remotely-sensed environmental factors significantly associated with the maximum presence of water bodies recorded on the ground, including 80 % of the observations for years 2008 and 2009 separately (multivariate logistic regressions with studied zone random effect are given - step 1).

|  | 2008-2009 | | | 2009-2010 | | |
| --- | --- | --- | --- | --- | --- | --- |
|  | 18,205 observations (30 zones) | | | 15,718 observations (30 zones) | | |
|  | Coef. | 95% CI* | p-value | Coef. | 95% CI* | p-value |
| **SPOT MNDWI rainy season (mean)** |  |  |  |  |  |  |
| Per 0.1 unit increase | 0.87 | 0.80 ; 0.94 | <0.001 | 1.95 | 1.83 ; 2.07 | <0.001 |
| **SPOT NDVI dry season (mean)** |  |  |  |  |  |  |
| Per 0.1 unit increase | 1.08 | 0.98 ; 1.18 | <0.001 | 0.90 | 0.81 ; 1.00 | <0.001 |
| **SPOT built-up areas** |  |  |  |  |  |  |
| Per 2.5m pixel increase | -0.11 | -0.13 ; -0.09 | <0.001 | -0.11 | -0.13 ; -0.09 | <0.001 |
| **DEM elevation** |  |  |  |  |  |  |
| Per meter increase | -0.21 | -0.24 ; -0.18 | <0.001 | -0.13 | -0.15 ; -0.10 | <0.001 |

* 95% confidence interval
