# Supplementary material for: Risk Mapping of Anopheles gambiae s.l. Densities Using Remotely-Sensed Environmental and Meteorological Data in an Urban Area: Dakar, Senegal
Source: PLoS One. 2012 Nov 30;7(11):e50674. doi: 10.1371/journal.pone.0050674 (PMC3511318; doi:10.1371/journal.pone.0050674)
Supplement: Table S3 — Remotely-sensed and ground meteorological and environmental factors significantly associated with the presence of An. gambiae s.l. larvae in the water samplings recorded on the ground, including 80% of the water bodies in 2008 and 2009 separately (multivariate logistic regressions with water body random effect are given - step 2). (DOC) [file pone.0050674.s003.doc]

Table S3. Remotely-sensed and ground meteorological and environmental factors significantly associated with the presence of *An. gambiae s.l.* larvae in the water samplings recorded on the ground, including 80% of the water bodies in 2008 and 2009 separately (multivariate logistic regressions with water body random effect are given - step 2).

|  | 2008-2009 | | | 2009-2010 | | |
| --- | --- | --- | --- | --- | --- | --- |
|  | 651 observations (59 water bodies) | | | 750 observations (75 water bodies) | | |
|  | Coef. | 95% CI* | p-value | Coef. | 95% CI* | p-value |
| **SPOT NDWI Mc Feeters dry season **** |  |  |  |  |  |  |
| Per 0.1 unit increase | 1.07 | 0.29 ; 1.85 | 0.007 | 0.95 | 0.05 ; 1.86 | 0.038 |
| **SPOT Soil BI dry season **** |  |  |  |  |  |  |
| Per 0.1 unit increase | 1.25 | 0.09 ; 2.41 | 0.035 | 0.66 | -0.27 ; 1.58 | 0.164 |
| **MODIS current night LST** |  |  |  |  |  |  |
| Per °C increase | 0.39 | 0.18 ; 0.61 | <0.001 | 0.11 | 0.00 ; 0.22 | 0.045 |
| **Ground rainfall amount in the preceding 30 days** |  |  |  |  |  |  |
| Per 10 mm increase | 0.04 | 0.01 ; 0.06 | 0.009 | 0.05 | 0.03 ; 0.07 | <0.001 |

* 95% confidence interval

** Mean in the water body and a 10-m ring around.
