# Supplementary material for: Risk Mapping of Anopheles gambiae s.l. Densities Using Remotely-Sensed Environmental and Meteorological Data in an Urban Area: Dakar, Senegal
Source: PLoS One. 2012 Nov 30;7(11):e50674. doi: 10.1371/journal.pone.0050674 (PMC3511318; doi:10.1371/journal.pone.0050674)
Supplement: Table S4 — An. gambiae s.l. larval productivity surrogate and environmental factors associated significantly with the An. gambiae s.l. HBR recorded on the ground, including all the observations for years 2008 and 2009 separately (negative binomial regressions are given - step 3). (DOC) [file pone.0050674.s004.doc]

Table S4. *An. gambiae s.l.* larval productivity surrogate and environmental factors associated significantly with the *An. gambiae s.l.* HBR recorded on the ground, including all the observations for years 2008 and 2009 separately (negative binomial regressions are given - step 3).

|  | 2008-2009 | | | 2009-2010 | | |
| --- | --- | --- | --- | --- | --- | --- |
|  | 406 observations (26 zones) | | | 408 observations (24 zones) | | |
|  | Coef | 95% IC* | p-value | Coef | 95% IC* | p-value |
| ***Anopheles* larval productivity surrogate **** |  |  |  |  |  |  |
| Per unit increase ** | 28.65 | 19.18 ; 38.12 | <0.001 | 20.30 | 8.95 ; 31.64 | <0.001 |
| **Built-up and asphalt mean surface ***** |  |  |  |  |  |  |
| Per m² increase | -1.15 | -1.71 ; -0.58 | <0.001 | -0.84 | -1.42 ; -0.25 | 0.005 |
| **Rainfall amount in the preceding 7 days** |  |  |  |  |  |  |
| Per 10 mm increase | 0.13 | 0.06 ; 0.21 | 0.001 | 0.18 | 0.09 ; 0.27 | <0.001 |

* 95% confidence interval

** Sum of (probabilities of presence of *Anopheles* larvae x surfaces of larval habitats in km²) for all water bodies contained in the 200-m buffer and 300-m to 1,000-m rings around the catching points, weighted by the distance to the catching point.

*** Weighted with distance to catching point (from 200-m buffer to 300-1,000-m rings).
